# ALD-R491 regulates vimentin filament stability and solubility, cell contractile force, cell migration speed and directionality

**DOI:** 10.3389/fcell.2022.926283

**Published:** 2022-11-22

**Authors:** Hyejeong Rosemary Kim, Samantha J. Warrington, Ana López-Guajardo, Khairat Al Hennawi, Sarah L. Cook, Zak D. J. Griffith, Deebie Symmes, Tao Zhang, Zhipeng Qu, Ying Xu, Ruihuan Chen, Annica K. B. Gad

**Affiliations:** ^1^ Department of Oncology and Metabolism, The Medical School, University of Sheffield, Sheffield, United Kingdom; ^2^ School of Biosciences, University of Sheffield, Sheffield, United Kingdom; ^3^ Aluda Pharmaceuticals, Inc., Menlo Park, CA, United States; ^4^ Cambridge-Su Genomic Resource Center, Medical School of Soochow University, Suzhou, China; ^5^ Madeira Chemistry Research Centre, University of Madeira, Funchal, Portugal

**Keywords:** vimentin, vimentin assembly, intermediate filaments (IFs), cytoskeletal organization, contractile forces, fluorescence recovery after photobleaching (FRAP), traction force microscopy (TFM), cell migration

## Abstract

Metastasizing cells express the intermediate filament protein vimentin, which is used to diagnose invasive tumors in the clinic. However, the role of vimentin in cell motility, and if the assembly of non-filamentous variants of vimentin into filaments regulates cell migration remains unclear. We observed that the vimentin-targeting drug ALD-R491 increased the stability of vimentin filaments, by reducing filament assembly and/or disassembly. ALD-R491-treatment also resulted in more bundled and disorganized filaments and an increased pool of non-filamentous vimentin. This was accompanied by a reduction in size of cell-matrix adhesions and increased cellular contractile forces. Moreover, during cell migration, cells showed erratic formation of lamellipodia at the cell periphery, loss of coordinated cell movement, reduced cell migration speed, directionality and an elongated cell shape with long thin extensions at the rear that often detached. Taken together, these results indicate that the stability of vimentin filaments and the soluble pool of vimentin regulate the speed and directionality of cell migration and the capacity of cells to migrate in a mechanically cohesive manner. These observations suggest that the stability of vimentin filaments governs the adhesive, physical and migratory properties of cells, and expands our understanding of vimentin functions in health and disease, including cancer metastasis.

## Introduction

The expression of the intermediate filament protein vimentin is a canonical marker of mesenchymal cells, and it is closely linked to their motile capacity (Liu et al., 2015; Messica et al., 2017). For example, the presence of vimentin is required for tissue regeneration, e.g., wound healing and aortic regeneration and directional cell migration (Schiffers et al., 2000; Cheng et al., 2016; Gan et al., 2016). Mesenchymal cell migration requires a dynamic cytoskeleton that can assemble and disassemble and thereby regulate cell mechanics and cell-matrix adhesions in a polarized and coordinated way. The adhesions exert force on the extracellular matrix, and this is required for the cells to move. Vimentin has been found to have key roles in these processes, including the regulation of cell shape, cell adhesion, cell motility, and organizing cellular mechanical forces (Mendez et al., 2010; Helfand et al., 2011; Havel et al., 2015; Liu et al., 2015; Costigliola et al., 2017; Hu et al., 2019; Vahabikashi et al., 2019). For more than half a century, the expression of vimentin has been widely used in the clinic to diagnose invasive carcinoma. Recent findings indicate that vimentin is not only a passive marker of carcinoma but may also promote tumor cell invasion (Wei et al., 2008; Vuoriluoto et al., 2011; Zhu et al., 2011; Chung et al., 2013; Rathje et al., 2014; Liu et al., 2016).

An increasing number of observations suggest that not only the total protein level of vimentin, but also the assembly of vimentin into filaments regulates cell adhesion, migration, and invasion. The assembly of intermediate filaments is a stepwise process in which the monomers first associate into dimers that then assemble into tetramers that form unit-length filaments (ULFs). ULFs then anneal longitudinally into intermediate filaments (Chernyatina et al., 2012; Danielsson et al., 2018).

Vimentin is not essential for the normal development of mice (Colucci-Guyon et al., 1994). However, it is required under pathological conditions, for example, during cancer metastasis, fibrosis and wound healing [as reviewed in (Eriksson et al., 2009; Danielsson et al., 2018; Ridge et al., 2022)]. Vimentin is therefore a promising drug target, because targeting vimentin could treat diseases without affecting the healthy cells, in which vimentin is not essential under physiological conditions (Colucci-Guyon et al., 1994). Small molecule compounds have been identified that interact, directly or indirectly, with vimentin. However, most of these compounds show low affinity to the target (Bollong et al., 2017; Trogden et al., 2018; Ramos et al., 2020; Strouhalova et al., 2020), and to date, the most investigated vimentin-targeting compound is Withaferin A which shows medium affinity to vimentin (Mirjalili et al., 2009; Rai et al., 2016; Dutta et al., 2019), and also binds to other molecules (Vanden Berghe et al., 2012). Therefore, there is a need to develop more specific targeting drugs. We have previously identified the chemical component ALD-R491 (R491), which was found to only bind to vimentin, in a screen of novel s-triazine compounds using photoaffinity labeling (Zhang et al., 2021). Computational modeling suggested that this compound binds to the amino acids, Arg273 and Tyr276, of vimentin (Zhang et al., 2021).

Many previous studies have analyzed the effects of overexpression of vimentin in cells of epithelial origin, which lack endogenous expression of vimentin. In contrast, this study uses oncogenically transformed, highly migratory and invasive human fibroblasts (Hahn et al., 1999; Evans et al., 2022). This cell type has a high endogenous level of vimentin, and has been well characterized with regard to the global transcriptome, and the nanoscale spatial distribution of vimentin, adhesions and cell migration (Hahn et al., 1999; Danielsson et al., 2013; Ronnlund et al., 2013; Evans et al., 2022). We have further found that the lamellipodia of these cells show non-filamentous vimentin variants of the size of ULFs, and that a pulse of these soluble vimentin variants precedes the formation of lamellipodia (Terriac et al., 2017). This is in line with the previous observations that local disassembly of vimentin induced lamellipodia formation (Helfand et al., 2011). In contrast, large, mature focal adhesions show assembly of vimentin filaments at their bases (Rathje et al., 2014; Terriac et al., 2017). We have previously also characterized the vimentin protein interactome in these cells. Therefore, the proteins that bind to vimentin in these cells are known (Evans et al., 2022). Taken together, these observations suggest that not only the total protein level of vimentin, but also the assembly of vimentin into filaments regulates cell adhesion, migration and invasion.

To clarify the role of vimentin assembly in the control of cell-matrix-adhesions and cell migration in these transformed fibroblasts, we analyzed the effect of R491 on transformed fibroblasts, with regard to the spatial organization of vimentin, the stability and assembly of vimentin filaments. In addition, we determined the effects of R491 on cell shape, cell protrusions, the cellular contractile force, as well as the effects on the cell migration speed, directionality and coherence during cell migration.

In this study, we found that treatment with the small chemical component R491 increases the soluble fraction of vimentin, and reduces the assembly and/or disassembly of vimentin filaments. This was accompanied by a spatial redistribution of vimentin filaments and reduced size of cell-matrix adhesions in cells, increased contractile forces, increased cell elongation, reduced cell migration speed, directionality, coherence and integrity during cell migration.

## Materials and methods

### Cell culture, DNA constructs and transfection

The transformed and metastasizing BjhtertSV40T-HRasV12 (BJ-Ras) cells were previously constructed by Hahn et al. by inserting three well-defined genetic elements: the telomerase catalytic subunit (hTERT) in combination with two oncogenes (the simian virus 40 large-T oncoprotein, and an oncogenic allele of H-ras) into neonatal human dermal fibroblast (Hahn et al., 1999). The cells were cultured as described previously (Gad et al., 2004). U87 cells were cultured in MEM containing 10% FBS as well as 50 U/ml penicillin and 50 μg/ml streptomycin in 10 cm dishes (Sigma). U118 cells were cultured in DMEM containing 10% FBS as well as 50 U/ml penicillin and 50 μg/ml streptomycin in 10 cm dishes. Transfection was performed by using Lipofectamine 2,000 (Invitrogen) according to the manufacturer’s instructions. For generation of stable Cas9-expressing cell lines, cells were transfected with the Cas9 vector. Stable transfectants were obtained by blasticidin selection. Then, cells were transfected with sgRNA vector followed by doxycycline treatment for 12 h. After 24 , the cells were selected for transduced cells using puromycin. Several single clones were chosen as the vimentin knockout cell lines which were confirmed by PCR and western blotting. Flag-tagged expression plasmids were constructed based on pCMV-Tag 2B (Stratagene). Full-length mouse vimentin cDNA was amplified from C57BL/6J mouse cDNA by RT-PCR and inserted into the vector by BamH1/Sal1, respectively. The vector was confirmed by sequencing. Amino acids were substituted in the vimentin (R273H; Y276F) by mutagenesis using the MutanBEST kit (TaKaRa) and confirmed by DNA sequencing. For FRAP analysis, the BJ-Ras cells were transiently transfected with a plasmid coding for vimentin-EGFP using jetOPTIMUS transfection reagent (Polyplus-transfection) following the manufacturer’s instruction 24 h prior to analysis (Rathje et al., 2014).

### CRISPR design and deletion of vimentin

For the human codon-optimized Cas9 expression, the doxycycline-inducible plasmid PB-TRE-NLS-linker-Cas9-ZF-IRES-hrGFP-Blasticidin was utilized. Two guide strands to target two segments in the human vimentin gene were designed using the CRISPR Design tool (http://crispr.mit.edu/) and ligated into the pGL3-U6-2sgRNA-ccdB-EF1a-Puromycin plasmid using a BsmBI restriction site.

### Transient knock down of vimentin

Vimentin knock down in BJ-Ras cells was performed with vimentin -targeting ON-TARGET siRNA-SMART pool (L-003551-00-0005, Dharmacon) or AllStars Negative Control siRNA (1027280, Qiagen) as control, using INTERFERin transfection reagents (101000028, Polyplus) according to the manufacturer’s protocol.

### Drug-treatment

The vimentin-binding compound used in this study was ALD-R491, (E)-1-(4-fluorophenyl)-3-(4-(4-(morpholine-1-yl)-6-styryl-1, 3, 5-triazinyl-2-amino) phenyl) urea. It has a molecular formula of C28H26FN7O2 and a purity of >98%. The compound was synthesized at Bellen Chemistry Co., Ltd. (Beijing, China), analyzed at Porton Pharma Solutions, Ltd. (Chongqing, China), and provided by Luoda Biosciences, Inc. (Chuzhou, China), and Aluda Pharmaceuticals, Inc. (CA, United States). The cells were treated with 5 μM R491 or DMSO for 4 h, if not stated otherwise in the figure legend. For each experiment, the control used contained DMSO at an equal concentration to the amount of R491 used.

### Live cell imaging

Live cell imaging was performed essentially as described previously (Evans et al., 2022). BJ-Ras cells were seeded at 10^3^ cells/well in the glass bottom 10 well slide chamber (Greiner Bio-One, 543079), and allowed to attach, spread and grow for 2 days. The cells were then treated with R491 or DMSO for 3 h, in combination with 0.05 μg/ml Hoechst to stain the nuclei. This was followed by live cell imaging, with images taken every 45 min for 17–23 h, using a 10 × magnification on a Zeiss Celldiscoverer7 Widefield Fluorescence Microscope (Carl Zeiss Microscopy, Thornwood, NY). For live cell imaging of siRNA transfected cells, the cells were grown for 3 days after the siRNA transfection to achieve a good KD efficiency, prior to the drug treatment and imaging. Analysis of 12 movies were performed for both the control and for the R491-treated cells for both live cell imaging experiments.

### Immunofluorescence staining and confocal microscopy

The cells were fixed and permeabilized in PBS containing 3.7% formaldehyde, 0.1% glutaraldehyde and 0.2% Triton-X100, and the immunofluorescent staining was performed as described previously (Gad et al., 2012a). The antibodies and an actin probe used are as follows: anti-mouse-vimentin, clone V9 (V6389, Sigma Aldrich), anti-rat-vimentin (MAB2105, R&D systems), anti-mouse-p-Tyr (sc-7020, Santa Cruz), anti-rabbit-β-tubulin (ab6046, Abcam), Alexa-Fluor-488 phalloidin (Invitrogen), Goat anti-mouse-Alexa-Fluor 555 (Invitrogen), Goat anti-rabbit-Alexa-Fluor 647 (Invitrogen) and Goat-anti-rat-Alexa-Fluor 647 (Invitrogen). The cells were then imaged with ZEISS LSM 980.

### Quantification of vimentin filament and cell-matrix adhesions

For quantification of vimentin filament distribution, the immunofluorescent images of control (n = 23) and treated cells (n = 20) were converted to greyscale using FIJI (v.1.52 g) and then analyzed with the FiNTA software (https://beta.finta-research.com/home, https://github.com/SRaent/FiNTA) (Flormann et al., 2021). We set the fiber diameter in the FiNTA software to 2 and 8 pixels for the control and treated cells, respectively, while all other settings were left as default. We chose these numbers because at these settings the FiNTA program traces the organization of vimentin filaments more faithfully than when we use the same diameter settings for control and drug treated cells. The loop diameters and circumferences were generated using the images of fiber network traces. The average fiber loop circumferences were then plotted as a scatter plot, each point representing a single cell. For quantification of vimentin filament thickness, immunofluorescent images of vimentin were converted to greyscale using FIJI (v.1.52 g). The fiber diameter was then quantified in the greyscale images, using the ‘graphical diameter calculation’ tool in the FiNTA software. In total, 15 control and 15 R491-treated cells were measured (5 from each biological repeat). After identifying the leading edge of the cell, 3 filaments per cell in the region between the leading edge and the nucleus were measured and average diameter per cell was calculated. To reduce bias, the filaments were chosen along a straight line in the region of interest. We could not measure the filament diameter in a totally blinded manner due to the apparent visual difference in the filament thickness between the control and R491-treated cells. For quantification of focal adhesion sizes, immunofluorescent phospho-tyrosine images were converted into greyscale and focal adhesions were highlighted using threshold tool in FIJI (v.1.52 g). Subsequently, the leading edge of the cells were selected and average focal adhesion size per cell was calculated (n = 7 each for control and R491-treated). Average focal adhesion sizes were compared using an unpaired t-test. The individual focal adhesion sizes were transferred to a prism file and the focal adhesion sizes were grouped into bins (bin width 1 μm^2^), and a frequency histogram was generated. Thereafter, we used a chi-square test to determine the statistical significance in the correlation between drug-treatment and focal adhesion size distributions.

### Fluorescence recovery after photobleaching

The Fluorescence recovery after photobleaching imaging and analysis were performed as previously described in Warrington et al., 2017 (Warrington et al., 2017). In brief, samples were imaged on an inverted Nikon A1 confocal microscope, with a Nikon 60 × 1.4 NA oil apochromatic objective lens. The images were taken at 1 frame per second, 30 gain, offset 0 with a pixel size of 80 nm, achieved using 5 x zoom, 512 × 512 pixels and with the pinhole at 1.2. For imaging a 488 nm diode laser was used to image at an output of 0.4%, with a band pass 505–550 nm filter. For bleaching the same laser was used at 30% and passed 2 times over a region of interest (ROI) −2 μm^2^ in size at a rate of 8 frames/second, with no image averaging. After bleaching, the fluorescence was 60%–80% of the initial fluorescence. Regions of vimentin near the nucleus were selected for analysis, we avoided regions near the cell periphery which often moved out of focus, as shown in Figure 2. For data acquisition, three pre-bleach images were captured, the area was bleached, followed by image-acquisition every 10 s. Data in which vimentin filaments moved out of focus were discarded.

### Cytoskeletal fractionation

The soluble and insoluble fractions of proteins were extracted as described previously (Rathje et al., 2014). In brief, to separate the soluble cytosolic fraction of the cells from the non-soluble filamentous fraction, the soluble fraction of the cells was extracted and the 2 cell fractions were analyzed using western blotting (Rathje et al., 2014). Similar extraction procedures have previously been shown to result in the extraction of vimentin tetramers (Soellner et al., 1985).

### Western blot

Proteins were separated using precast polyacrylamide gels (4%–15%) (4568084, Bio-Rad Laboratories Ltd.), and western blot procedures were performed as described previously (Evans et al., 2022). Immunoprecipitation was performed as previously described (Qu et al., 2016). The list of antibodies used is as follows: anti-mouse-vimentin (V6389, Sigma Aldrich), anti-rabbit-β-tubulin (ab6046, Abcam), anti-mouse-β-actin (A-1978, Sigma Aldrich) and anti-mouse-FLAG (Sigma Aldrich, F3165). HRP-conjugated goat anti-mouse/rabbit secondary antibodies (GtxRb-004-DHRPX/GtxRb-003-DHRPX, Immunoreagents). For vimentin monomers/tetramers detection, 20–50 μg of total protein extracts were loaded on the gel. When detecting the vimentin tetramer, β-Mercaptoethanol was not added into the sample buffer. The NIH ImageJ software (version 2.3.0/1.53f) was used for the quantification of the bands from the western blots.

### Cell shape and migration analyses

Cell shape was quantified using FIJI (v.1.52 g), a scale bar was used to calibrate images at 2, 7, 12, and 17 h where all cells in the frame with a clear border were traced manually with the freehand draw tool allowing measurement of area and shape descriptors; circularity and aspect ratio. For cell shape analysis, the number of cells (indicated as treated/control) were as follows, 2 h (219/284), 7 h (195/285), 12 h (176/256) and 17 h (176/223). Migration speed and persistence of DMSO (n = 394) and R491-treated cells (n = 318) was tracked using the FIJI (v.1.53c) TrackMate plugin, by calibrating to 1.1:1 pixels: µm, setting the frame interval to 45 min and using these settings: Difference of Gaussian (Dog); linking max. distance, 200 µm. The estimated object size threshold were adjusted to track the majority of the cells. All other settings remained in the default setting.

### Traction force microscopy

Traction force microscopy experiments were performed using collagen coated 12 kPa hydrogels with 0.2 μm Yellow/green fluorospheres Matrigen SoftTrac™ plates (Cellguidance-Cambridge). Plates were equilibrated with cell culture medium for 30 min at 37°C. BJ-Ras fibroblast cells were seeded at 2500 cells/cm^2^ and allowed to adhere for 1 h prior to 4 h drug treatment and imaging. Phase images of attached cells and fluorescent embedded beads were collected using 40 x magnification on a Zeiss Celldiscoverer7 Widefield Fluorescence Microscope (Carl Zeiss Microscopy, Thornwood, NY). Images of the fluorescent microspheres on a relaxed hydrogel were collected after 5 min treatment with a 0.5% (w/v) Triton-X-100, 20 mM NH_4_OH PBS solution to remove cells. Treatment with Triton-X100 is an efficient method to detach cells (Franco-Barraza et al., 2016; Teo et al., 2020). Displacement of beads before and after the removal of cells was tracked by particle imaging velocimetry followed by Fourier transform traction cytometry to estimate the corresponding cell traction force field, using a modified ImageJ macro kindly shared by Dr. S. Lee (Lee and Kumar, 2020) from a previously described method (Martiel et al., 2015). The total traction forces (in N) were measured by integrating traction forces over the cell area. The elastic energy (in J) stored in the gel to produce the observed deformation was calculated by summing the products of displacement with the force over the cell area.

### Counting of cells that exhibit the “tail phenotype”

Timelapses obtained as described in the Live-cell imaging section above were analyzed with regard to the number of cells which leave their tips behind during cell migration. For this, two assessors analyzed independently 11 control- and 11 R491-treated timelapses, by manual tracing frame-by-frame of 480 control-treated or 454 R491-treated cells. Cells partially out of frame or too crowded together and overlapping were excluded from the analysis.

### Statistical analysis

Statistical analysis of focal adhesion sizes was performed using unpaired t-test in GraphPad Prism (v. 9.4.0). The focal adhesion sizes and frequencies were used to generate a histogram (bin size, 1 μm^2^). The statistical analysis of western blots was performed using GraphPad Prism (v.6.0). Data were presented as mean ± SD or mean with SEM. Differences between groups were analyzed by Anova and Student’s t-test. *p* ≤ 0.05 was considered statistically significant. The mean values of the aspect ratio, cell migration speed and persistence, cells from which debris is detached and left behind during migration, filament distribution, as well as Traction force microscopy data were analyzed using GraphPad Prism (v.9.3.1). And statistical differences were assessed using an unpaired two-tailed parametric t-test or a Mann-Whitney U test when the results were non-parametric. Results were presented as mean ± SD, and a *p* ≤ 0.05 was considered significant. For the Fluorescence recovery after photobleaching analysis, once processed in NIH ImageJ FIJI software (v.1.53f), the data was transferred to GraphPad Prism (v.9), plotted on an X-Y graph and a one-phase exponential association curve fitted. An extra sum-of-squares F test was performed to compare the plateau and the rates for the control and treatment conditions. To do this the best fit values were obtained from the fitted curve for the plateau (Ymax), the rate (K) and the half-life. Then, the plateau (Ymax), the rate (K) parameters were individually compared to a simpler model whose parameters are shared among the data sets compared. The null hypothesis for this test is that the two samples come from the same population and so share their parameters.

## Results

### R491 causes a more bundled and disorganized distribution of vimentin filaments

**FIGURE 1:**
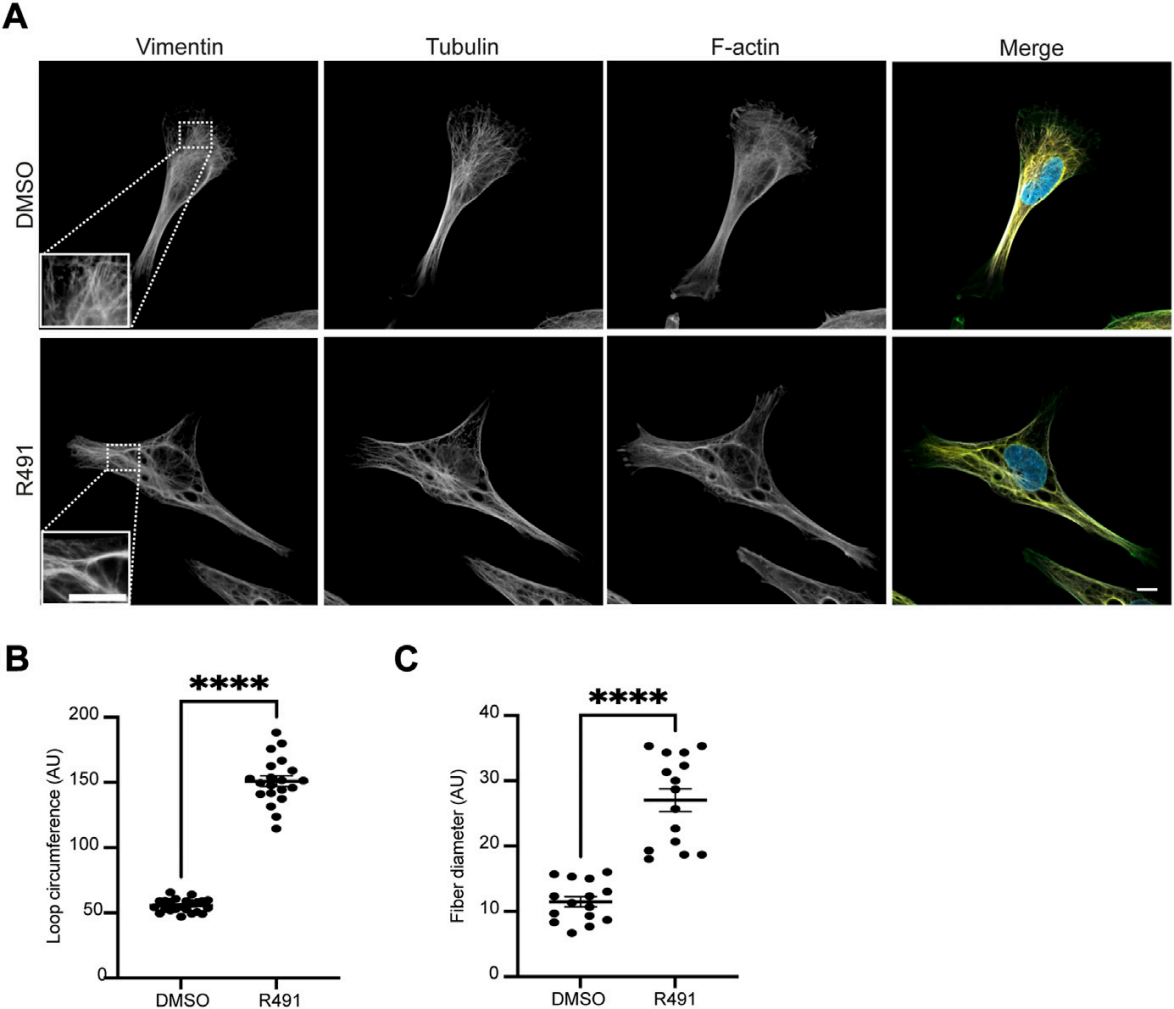
R491-treatment changes the spatial organization of vimentin filaments. **(A)** Representative images of BJ-Ras-cells with 5 μM control (top) or R491 (lower panel), showing vimentin, microtubule and actin, as indicated, and merged images of vimentin (red), F-actin (green), tubulin (white) and nuclei (blue). Inset images: magnified views of boxed areas in corresponding images. Scale bars: 10 μm. The graphs show the quantification of **(B)** the loop circumferences of vimentin filaments in DMSO (*n* = 23) and R491-treated (*n* = 20) cells, and **(C)** the diameter of filaments in DMSO (*n* = 15) and R491-treated (n = 15) cells. ****p ≤ 0.0001. AU: arbitrary unit. Data presented as mean ± SEM.

The changes in vimentin filament distribution can affect cell migration speed and directionality (Ronnlund et al., 2013; Rathje et al., 2014; Danielsson et al., 2018). Therefore, we aimed to determine how the R491-treatment would affect the distribution of vimentin filaments. To this end, we analyzed the organization of vimentin IFs in BJ-Ras cells, as described in the Materials and Methods section. We observed that in the R491-treated cells, the vimentin filaments were distributed in a more disorganized manner throughout the cytoplasm, as compared to the control (Figure 1). In the control cells, the vimentin filaments radiated out from the perinuclear position to the leading edge of the cells in an organized and more parallel arrangement. In contrast, the vimentin filaments in the R491-treated cells were more disorganized with increased loop circumferences and in thicker filament bundles (Figure 1).

### R491 increases the stability of vimentin filaments with no change in the rate of the delivery of vimentin to the filaments

Vimentin filaments are dynamic, with constant exchange of subunits, as previously shown using FRAP analysis ((Yoon et al., 1998), Figure 2). Because R491 binds vimentin at the ^273^RxxY motif and inhibits the formation of vimentin tetramers in U87 and U118 cells [(Zhang et al., 2021), Supplementary Figure S1], we hypothesized that the drug would change the vimentin filament dynamics. Using FRAP analysis, we observed that vimentin filaments in BJ-Ras cells treated with R491 at 5 µM or 10 µM for 3 h prior to imaging showed increased stability but showed no change in the rate of fluorescence recovery as compared to the controls (Supplementary Figure S2; Supplementary Table S1). No change was observed with 1 µM concentration of the drug (Supplementary Figure S2). Taken together, this suggests that the drug increases the stability of vimentin filaments in the cell not by changing the rate of delivery of vimentin subunits to the filaments but by altering the filament subunit assembly and/or disassembly dynamics.

**FIGURE 2:**
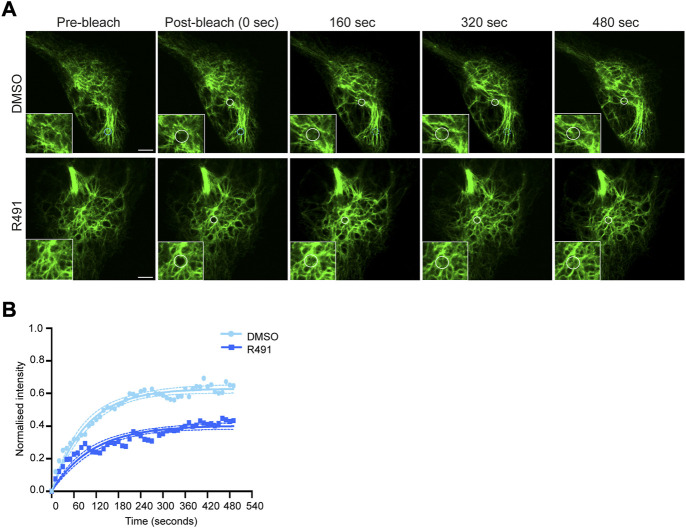
R491-treatment stabilizes vimentin in BJ-Ras cells. **(A)** Representative FRAP images of cells treated with 5 µM control or R491 3 h prior to imaging. Inset images show magnified views of the bleached areas in the larger images. The FRAP-bleached regions are shown in white circles over time. The blue circle in the top panel indicates a FRAP-bleached region near the cell periphery, in which vimentin filaments moved and therefore were difficult to track (dotted blue circles), and excluded from the data set. **(B)** FRAP recovery curves of vimentin-EGFP in cells treated with 5 μM control (light blue, *n* = 14) or R491 (dark blue, *n* = 13), showing the means of at least 3 biological repeats. One-phase exponential recovery curve was fitted (solid line), and 95% confidence intervals are shown (dotted lines). ****p ≤ 0.0001 drug treated compared with control plateau (Ymax). *p* = 0.319 for drug-treated as compared with control Rate of recovery (K). Scale bar: 5 µm.

### R491 increases the soluble fraction of vimentin

**FIGURE 3:**
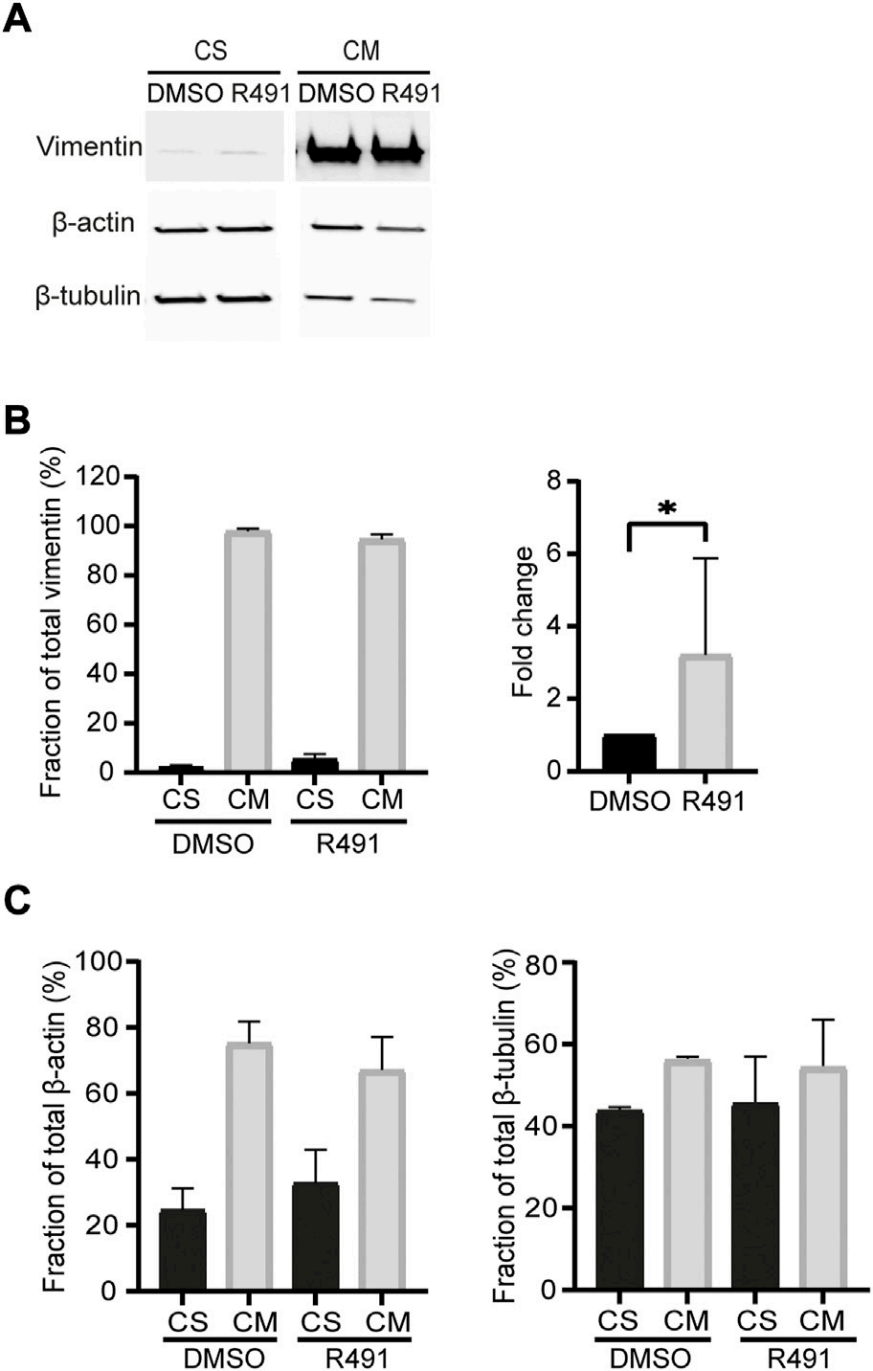
R491-treatment increases the soluble fraction of vimentin in BJ-Ras cells. **(A)** Representative western blot images of cells with DMSO control- or R491-treatment, analyzed for vimentin, actin and microtubules in soluble (CS) and insoluble (CM) cytoskeletal fractions, as quantified in **(B**,**C)**. The western blot images are taken from the same membrane. Graphs show means of at least 3 biological repeats. Data presented as mean ± SEM. *p < 0.05.

In migrating cells, the ways in which different assembly statuses of vimentin are distributed affect the shape, polarity and motility of cells (Helfand et al., 2011). Non-filamentous vimentin is shown to be enriched within the lamellipodia, whereas mature filaments inhibit lamellipodia formation. As R491 reduces the production of tetramers and also the mobile fraction of vimentin (Supplementary Figure S1, Figure 2), we speculated that the assembly and incorporation process of ULFs into the mature filament might be affected by R491 treatment, and we would expect an increase in the amount of soluble vimentin. We therefore isolated and quantified the fold change of the vimentin soluble fraction in BJ-Ras cells after drug treatment. In line with previous reports, the soluble fraction of vimentin in fibroblasts was much lower than that of actin or tubulin [(Rathje et al., 2014), Figure 3]. However, there was an increased soluble fraction of vimentin in R491-treated cells, with an average 3.2-fold increase, as compared to the control. R491-treatment resulted in a slight, but not significant, trend of increased soluble fractions of actin and tubulin (Figure 3).

### R491 decreases the speed and persistence of migrating cells

**FIGURE 4:**
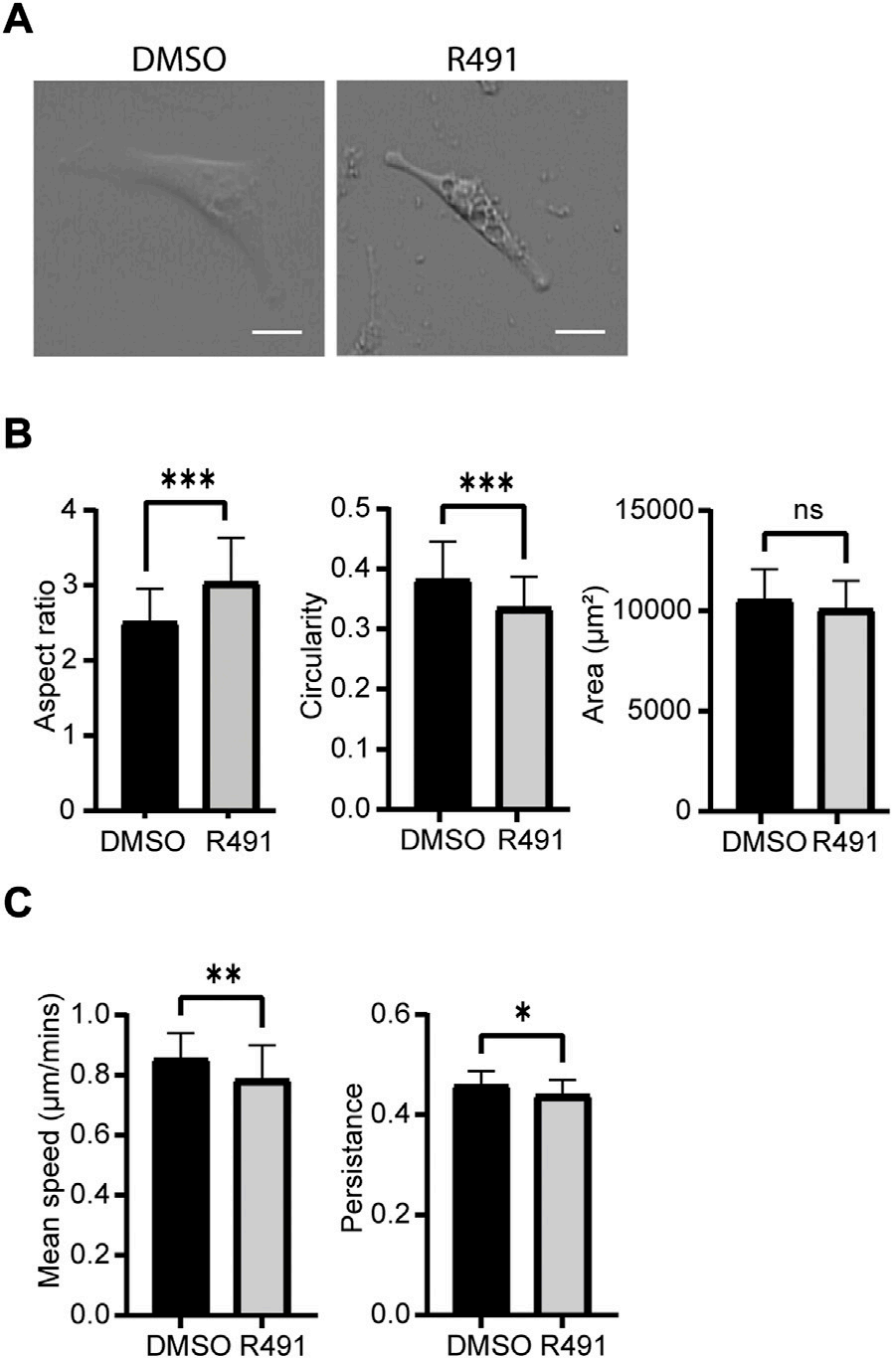
R491-treatment decreases the speed and directionality of cell migration and increase cell elongation in BJ-Ras cells. **(A)** Oblique-contrast images of BJ-Ras-cells treated with control or R491, as indicated, and quantified with regard to **(B)** aspect ratio, circularity, spreading area or **(C)** migration speed and persistence, as indicated. Graphs show means of at least 3 biological repeats. Data presented as mean ± SD. *p ≤ 0.05, **p ≤ 0.01, ***p ≤ 0.001. Scale bars: 50 µm.

**FIGURE 5:**
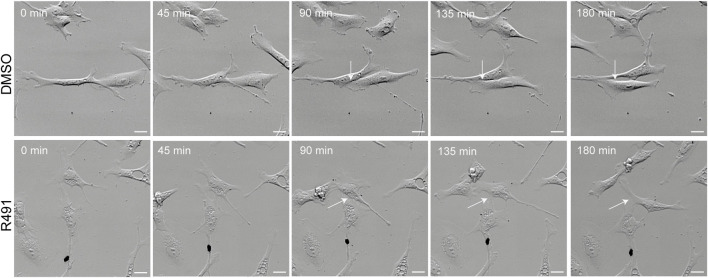
R491-treated cells migrate past other cells in a non-coordinated manner with lamellipodia-like protrusions. Montage of 5 sequential images (time frame of 45 min) of a movie showing representative control- or R491-treated BJ-Ras cells, as indicated. White arrows indicate the protrusions between cells as they migrate. Scale bars: 50 μm.

**FIGURE 6:**
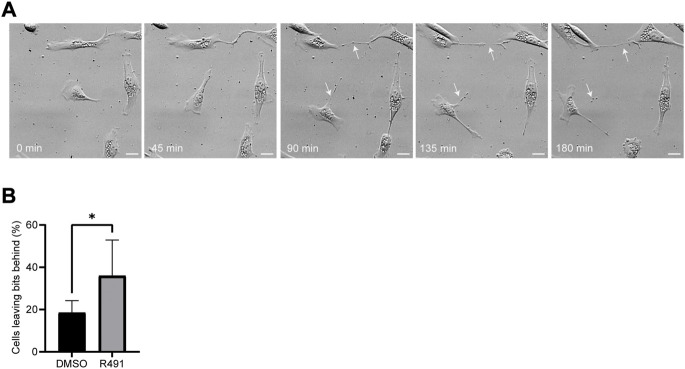
R491 forms long thin extensions at the training edge in BJ-Ras cells that often detach during cell migration. **(A)** Montage of 5 sequential images of representative R491-treated cells. White arrows indicate the retraction fibers that remain behind the cell, and often break off, during migration. **(B)** Fraction of cells from which debris is detached without and with R491-treatment, as indicated. Data presented as mean ± SD. **p ≤ 0.01. Scale bars: 50 μm.

Cell motility is an essential part of cancer cell metastasis and it depends on the cytoskeleton and cell-ECM adhesion. Since the distribution of vimentin was changed in response to R491-treatment, we analyzed if R491 also changed the adhesion, migration and shape of BJ-Ras cells. At all timepoints analyzed, there was a trend to decreased cell migration speed and directionality, and an increase in cell elongation of the R491-treated cells, as compared to control, with significant differences at the 17 h time point (Figure 4, Supplementary Figure S3). The R491-but not control-treatment resulted in an erratic formation of lamellipodia in all regions at the cell periphery during cell migration, and a non-coordinated movement of the different parts of the cells (Figure 5). The drug also resulted in very thin and long trailing edges of migrating cells which often broke off from the cell body during migration, leaving the rear end of the cell behind and attached to the underlying surface (Figure 6). In multi-cell clusters, the control-treated cells moved past each other in a flat and elongated shape, apparently adapting to the adjacent cells, while the R491-treated cells did not elongate, or adapt their shape. Instead, the drug-treated cells passed by other cells without a clear leading edge, and with flat, lamellipodia-like protrusions forming simultaneously in different directions (Figure 5). To determine if the effect of R491 on cell motility is due to an interaction with vimentin, we analysed the migration of vimentin knockdown cells, in the presence of drug or control. We observed no effect of the drug in these cells, suggesting that the effect of the drug depends on vimentin (Supplementary Figure S4).

### R491-treatment increases the traction force of single cells

**FIGURE 7:**
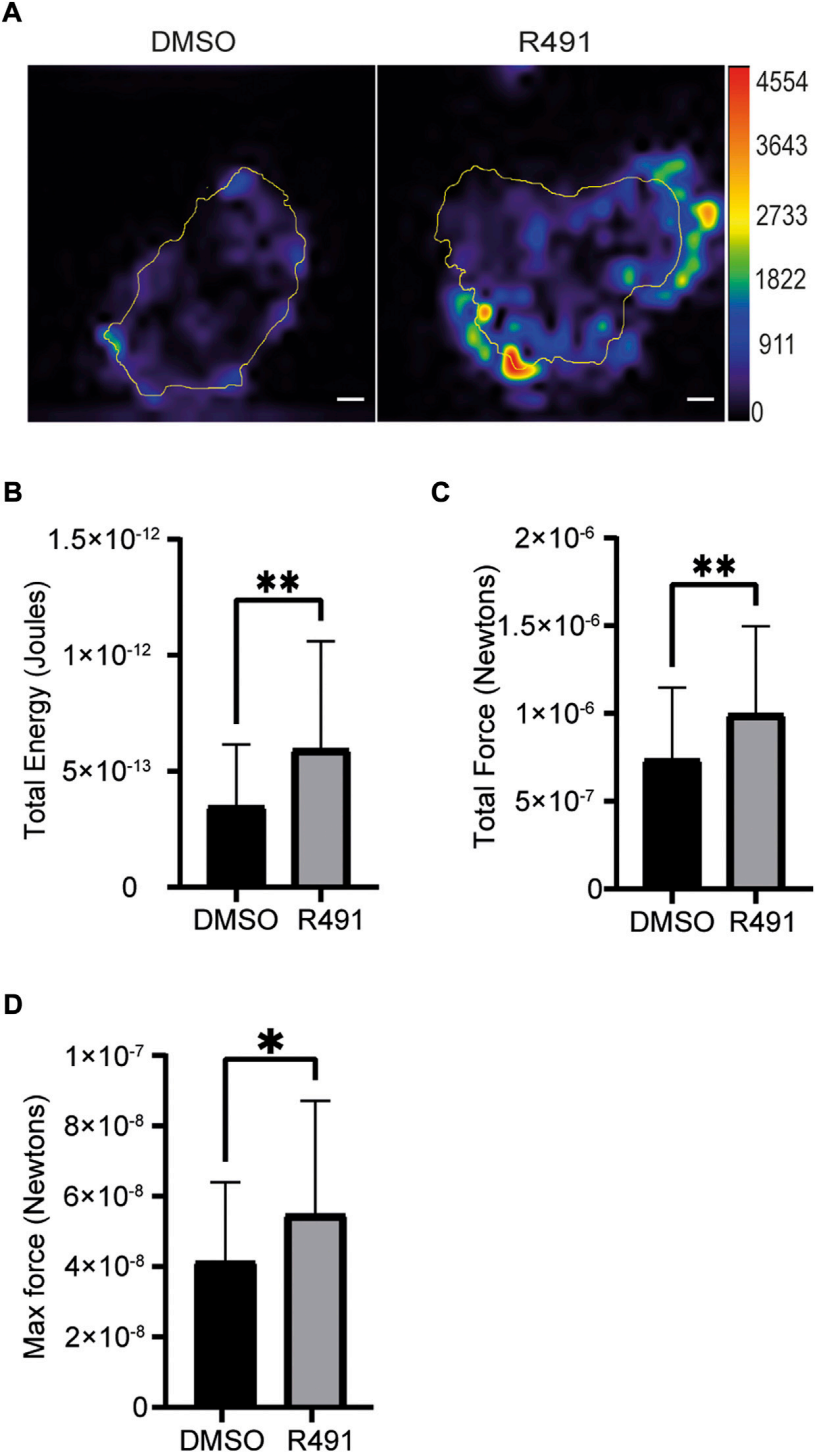
R491-treatment in BJ-Ras cells induces cellular contractile forces. **(A)** Representative traction force maps of control- and R491-treated cells with the color showing the magnitude of traction force (in pascals), as indicated in the color key. Quantification of **(B)** the total traction forces (traction forces integrated over cell area) exerted by the cells, **(C)** the total strain energy and the **(D)** maximum force is shown, as indicated. Traction force microscope measurements were taken from 5 µM DMSO- or R491-treated cells, from three biological repeats, *n* = 55 and *n* = 42 cells respectively. Data presented as mean ± SD. *p ≤ 0.05, **p ≤ 0.01. Scale bars: 10 µm.

Cross talk between vimentin and other cytoskeletal proteins has been observed to regulate the contractile forces of cells (Eriksson et al., 2009; Liu et al., 2015). To determine if R491-treatment had an effect on the contractile forces of single cells, we compared the forces between drug-treated and control BJ-Ras cells. We observed that the R491 increased the total contractile force, stored elastic energy and maximum force of cells, as compared to the control (Figure 7).

**FIGURE 8:**
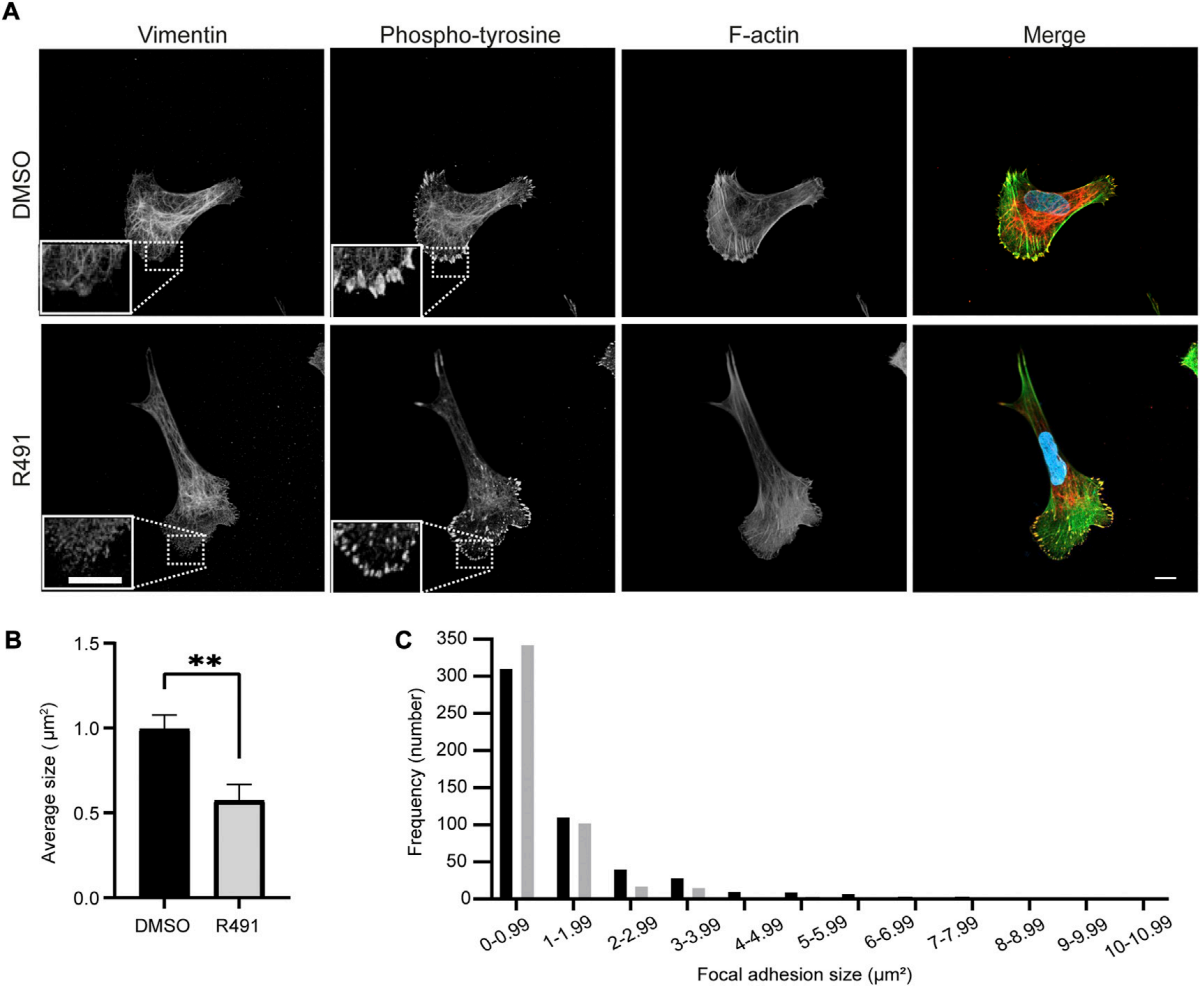
R491 increases the fraction and number of small focal adhesions. **(A)** Representative confocal microscopy images of BJ-Ras cells treated with 5 μM control or R491, showing vimentin, phospho-tyrosine, F-actin, as indicated, and merged images with vimentin (red), phospho-tyrosine (yellow), actin (green) and nuclei (blue). Inset images: magnified views of boxed areas in the larger images. Scale bars: 10 μm. The graphs show **(B)** the average size of cell-matrix adhesions/per cell, with n = 516 and 478 focal adhesions for control- and R491-treated cells, respectively, and **(C)** the frequency distribution of the binned sizes of focal adhesions of control- (black) or R491- (grey) treated cells. **p ≤ 0.01. The difference in the frequency distribution was significant with *p* ≤ 0.0001.

To understand the basis for the drug-dependent effect on the contractile forces of cells, we compared the size and numbers of cell-matrix focal adhesions at the leading edges between R491-treated and control-treated BJ-Ras cells. We found that R491-treatment decreased the average size of the focal adhesions. We also observed and there was a significant increase in the number of small focal adhesions in the R491 treated cells when compared to control-treated cells (Figure 8).

## Discussion

In this study, we found that the vimentin-targeting small chemical compound R491 changes the cell-matrix adhesions, the cellular contractile forces, the cell migration and the cell shape of oncogenically transformed fibroblasts *via* the disruption of vimentin filament dynamics.

The other drugs known to target vimentin, such as Withaferin A and FiVe1 induce a collapse of vimentin filaments towards the perinuclear area *via* vimentin phosphorylation (Thaiparambil et al., 2011; Bollong et al., 2017). However, R491 causes more subtle changes in the spatial distribution of vimentin filaments, i.e., an increase of bundled and disorganized filaments. The vimentin is constantly exchanged between the mature vimentin filaments and the soluble pool of vimentin (Noding et al., 2014; Danielsson et al., 2018). We observed that R491 reduces this dynamic exchange, and increases the stability of the vimentin filaments. There was no change in the rate of the delivery of vimentin into the FRAP-bleached area, which indicates that R491 interferes with the incorporation of vimentin subunits into the mature filaments and/or reduces the loss of vimentin subunits from the mature filaments. Because the drug also reduced the pool of vimentin tetramers in cells, it seems reasonable to assume that R491 inhibits the incorporation and assembly of vimentin into mature filaments, and thereby, indirectly, prevents the loss of vimentin from filaments. An increased filament stability might be expected to reduce the soluble vimentin fraction. In contrast, we observed an increased soluble fraction of vimentin in the drug-treated cells. These apparent contradictory observations could be explained if R491 binds to vimentin monomers and tetramers and thereby suppresses the formation of ULFs required for filament assembly (Danielsson et al., 2018; Mucke et al., 2018). If there is an extensive pool of ULFs in the cells, this reduced formation of vimentin ULFs would not necessarily delay the filament assembly. It is possible that a reduced pool of ULF could not be detected in the soluble vimentin fraction, if ULFs are not extractable in our experimental conditions. This is in line with our previous observations that most non-filamentous vimentin fragments in these cells have the expected length of ULFs (Terriac et al., 2017), also similar extraction procedures have been shown to result in the extraction of tetramers (Soellner et al., 1985). Alternatively, R491 may bind to mature vimentin filaments and inhibit their disassembly, independently of vimentin assembly. In addition, the bundling of vimentin filaments may hinder the process of phosphorylation and disassembly of mature vimentin filaments, and thereby stabilize the filaments. It is possible that the amount of vimentin filaments in the cell is rigorously controlled, to the extent that if the cell cannot break down the large bundles of filaments, it will not assemble new vimentin filaments, which would keep the net amount of filaments constant. The ULF would be assembled, but not added to existing fibres, and instead remain in, and increase the soluble pool of vimentin. Further studies are required to determine how the drug stabilizes vimentin filaments.

We observed that treatment with R491 increased the contractile force of cells. Although we do not exclude alternative mechanisms of action, the increased contractile force, together with our earlier observation that vimentin is the only protein that R491 binds to, suggests a key role for vimentin in the regulation of contractile forces. Vimentin has been reported to regulate the traction forces that cells exert on their environment. Jiu et al., showed that the loss of vimentin increases cellular contractile forces, by activation of GEF-H1 followed by RhoA activity and actin stress fiber formation (Jiu et al., 2017). We therefore speculate that R491 increases these forces by blocking the interaction between GEF-H1 and vimentin, leading to RhoA activation. However, we do not exclude alternative mechanisms of action. The increased force in the drug-treated cells was accompanied by an increase in the numbers of small, but not of large cell matrix adhesions. This is in line with the previous observations that small nascent cell-matrix adhesions are linked to higher contractile force than large and mature adhesion (Beningo et al., 2001; Gad et al., 2012b). Our experiments suggest that an increased stability of vimentin filaments induces contractile forces, and that these forces also can be increased by the soluble fraction of vimentin. It is important to note that R491-treatment does not eliminate the vimentin (Li et al., 2021; Wu et al., 2021). Taken together, these observations suggest that it is the dynamic turnover of vimentin filaments and/or their solubility, and not the presence of vimentin filaments *per se* that regulates the force exerted over cell-matrix adhesions. In contrast to the observations presented above, vimentin has also been shown to induce cellular contractile force (Eckes et al., 1998; Gregor et al., 2014; Liu et al., 2015). We propose that the discrepancies between these studies could be due to distinct molecular mechanisms regulating the contractile force in different types of cells, and/or in cells adhered to different extracellular matrix components. Taken together, these observations highlight the need for future studies on the role of vimentin in the regulation of cellular contractile forces.

In agreement with the key role for vimentin filament dynamics in cell migration, we observed that R491 decreased the speed and persistence of cell migration. This is consistent with our previous observation that R491 reduces the collective migration of epithelial cancer cells (Wu et al., 2021). The formation of a protruding lamellipodia at the cell front is essential for 2D cell migration. Mature vimentin filaments have previously been shown to inhibit, and/or soluble vimentin to promote lamellipodia formation (Helfand et al., 2011). When the cell changes direction, the lamellipodia at the cell front stops to ruffle, and a new lamellipodia emerges in the future direction of movement (Abercrombie, 1961). Therefore, we speculate that the defective and un-coordinated cell migration we observed is due to an increased soluble pool of vimentin which leads to an erratic formation of lamellipodia that causes frequent changes of the direction of cell movement which slows down the speed and reduces the directionality of cell migration. Although, we do not exclude the possibility that the soluble fractions of actin and/or tubulin can contribute to this phenotype, we did not observe significant changes to the soluble pool of actin or tubulin in our experimental conditions.

The reduced cell migration speed and directionality in the R491-treated cells were accompanied by a more elongated cell shape, and long trailing edges of cells that showed a tendency to break off during the cell migration. This is in contrast to the common finding that more elongated cells show increased cell migration speed and directionality, for example, when cells undergo epithelial to mesenchymal transition. Vimentin organize the spatial distribution of organelles within the cytoplasm (Guo et al., 2013). The defects in cell migration could therefore be due to defective organization of organelles in cells. The retraction of trailing edge is a rate limiting factor of the cell migration (Palecek et al., 1998). It is therefore likely that the drug inhibits a vimentin-dependent coordination of the physical and adhesive properties of the leading and trailing edges of the migrating cells. In particular, increased lamellipodia formation and contractile forces at the leading edges would drive the cell front forward, whereas the retraction of the trailing edges would be inhibited without functional vimentin. The defective retraction of the trailing edge could also be a consequence of more stable focal adhesions at the rear of drug-treated cell, possibly by reduced disassembly and turnover of focal adhesions. As microtubules stimulate focal adhesion turnover, and use vimentin filaments as templates for growth (Gan et al., 2016), it is possible that R491 reduces the capacity of vimentin to facilitate microtubule-dependent disassembly of the focal adhesions (Kaverina et al., 1999; Rid et al., 2005). Defective vimentin organization can also result in loss of the mechanical cytoplasmic coherence required for the retraction of the trailing edge. Therefore, we speculate that an enhanced stability of vimentin filaments and an increased soluble pool of vimentin reduces the capacity of cells to coordinate the cytoskeleton, the mechanical properties and cell-matrix adhesions required for an efficient cell migration. The elevation of cellular contractile forces is usually accompanied by loss of cell elongation and a rounder cell shape. In contrast, we observed that R491 caused both more elongated cells and increased contractile forces. In line with earlier observations, we speculate that the elevated contractile forces are due to an increased lamellipodia formation (Beningo et al., 2001; Gad et al., 2012b), while cell elongation is a consequence of increased lamellipodia-based forces at the front, combined with a defective retraction of the trailing end of cells.

Taken together, our observations suggest that spatial and temporal regulation of the dynamics of vimentin filaments is required for the speed, the directionality and the mechanical coherence of cells during cell migration. It further supports the concept that the soluble and filamentous pools of vimentin have fundamentally different functions in cell mechanics, adhesion and migration. In future studies, it would be interesting to determine how R491 regulates the formation of lamellipodia and focal adhesions using live cell imaging and super resolution microscopy such as STED. How the drug changes the mechanical properties of assembly and disassembly of the vimentin filaments should be also be determined.

In conclusion, we present here a characterization of the effects on cells of the vimentin-targeting compound ALD-R491. The new understanding of how the dynamics, solubility and spatial organization of vimentin are linked to cellular contractile forces, cell adhesions, cell migration and cell shape contributes to our understanding of vimentin functions in health and disease, including in cancer metastasis.

## Data Availability

The original contributions presented in the study are included in the article/Supplementary Material, further inquiries can be directed to the corresponding author.
