# Supplementary material for: ALD-R491 regulates vimentin filament stability and solubility, cell contractile force, cell migration speed and directionality
Source: Front Cell Dev Biol. 2022 Nov 22;10:926283. doi: 10.3389/fcell.2022.926283 (PMC9723350; doi:10.3389/fcell.2022.926283)
Supplement: Supplementary file 1 [file DataSheet1.PDF]

# *Supplementary Material*

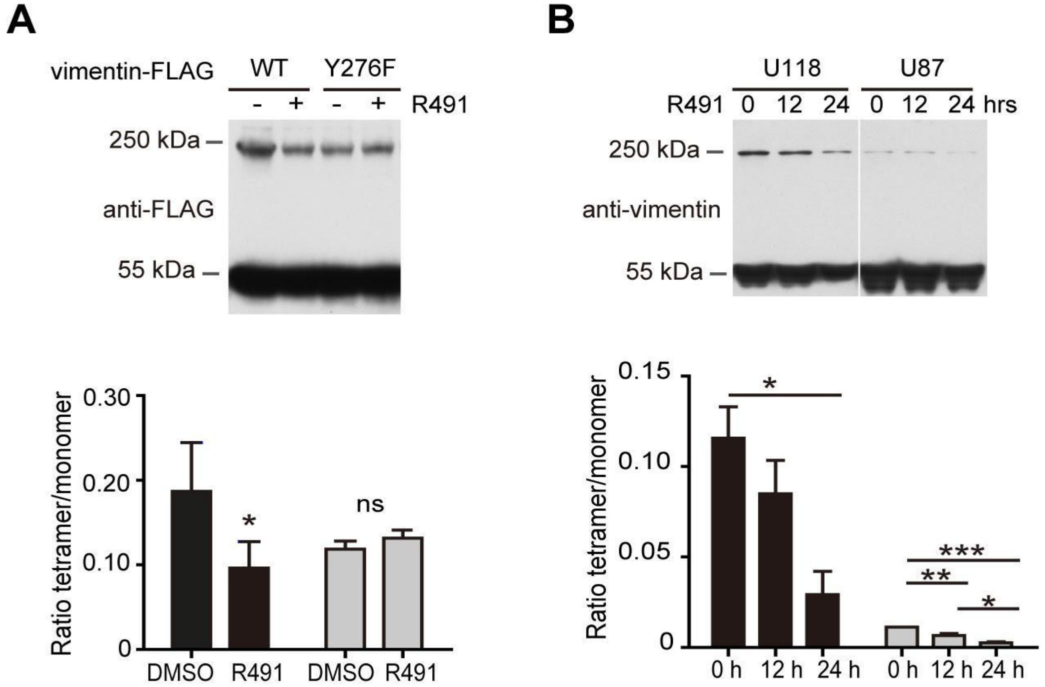


**Supplementary Figure 1.** R491 decreases the formation of vimentin tetramers. **(A)** Western blot image showing vimentin molecular weights in wild type and Y276F mutated U87 cells treated with 10 μM R491 or DMSO control, as indicated. Quantifications shown in lower panel with wild type U87 (black) and U87 Y276F mutated (grey). **(B)** U118 (black) and U87 cells (grey) treated with R491 for different periods showing vimentin, as indicated in top panel, with lower panel showing quantifications. Data represents means between cells from at least 3 independent experiments. *P≤0.05; **P≤0.01; ***P≤0.001 vs. relevant controls.

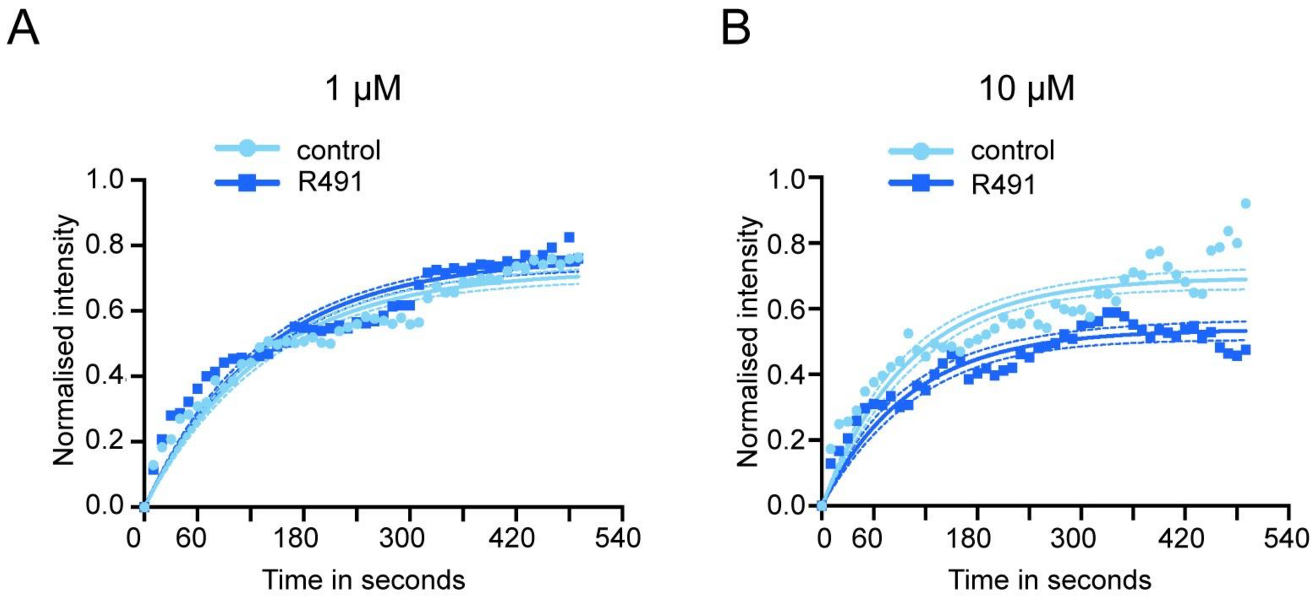


**Supplementary Figure 2**. R491-treatment stabilizes vimentin in BJ-Ras cells. FRAP recovery curves of vimentin-EGFP in regions located near the nucleus in mock treated with DMSO (light blue) and with different concentrations of R491 (dark blue) **(A)** 1 µM R491 (n=23) and DMSO (n=21), and **(B)** 10 µM R491 (n=15) and DMSO (n=17). Graphs show means of at least 3 biological repeats. One-phase exponential recovery curve was fitted (solid line), and 95% confidence intervals are shown (dotted lines). Compared drug and control plateau (Ymax) for **(A)** P=0.337, and **(B)** ***P≤0.0001. Compared the drug and control Rate (K) of recovery **(A)** P=0.639, and **(B)** P=0.132).

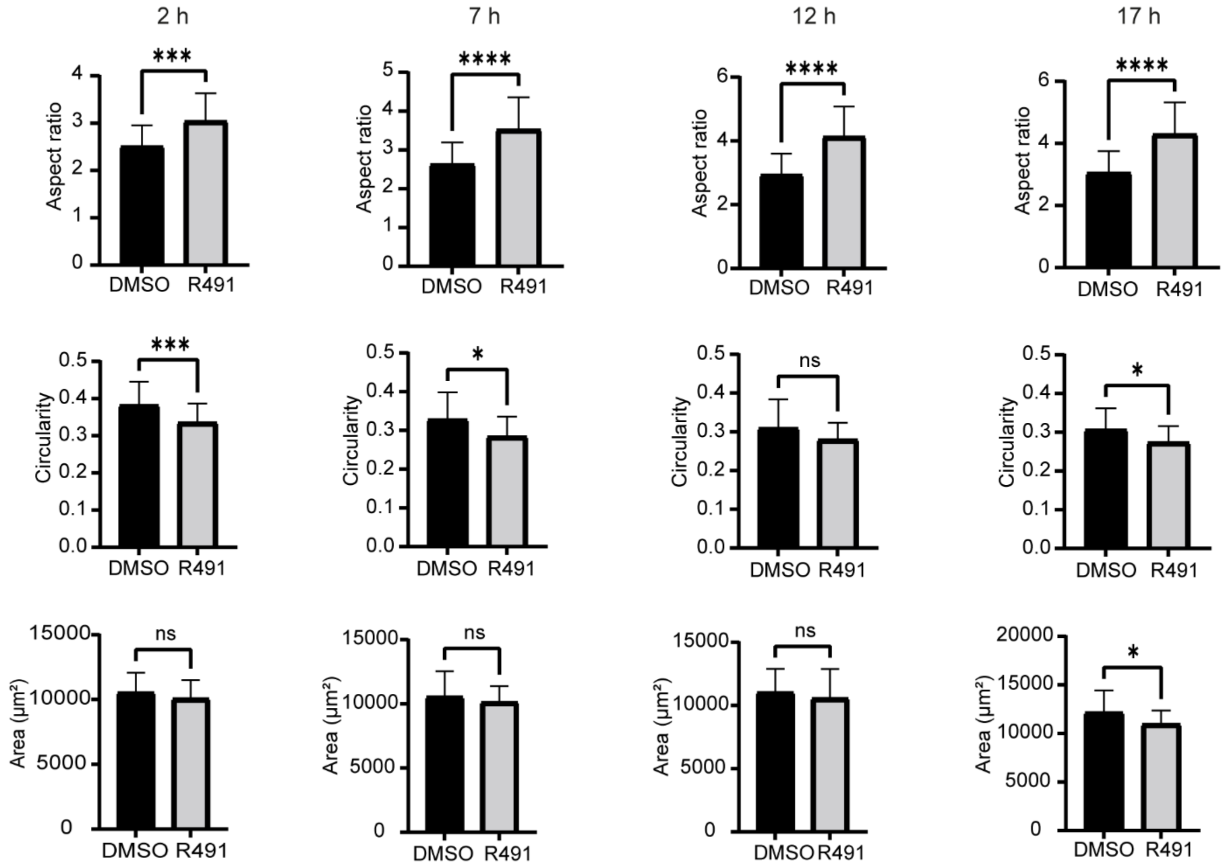


**Supplementary Figure 3.** R491 increases the aspect ratio, reduces circularity and the spreading area of the cells. Bj-Ras cells without (black) and with (grey) R491 with regard to aspect ratio (top panel), circularity (middle panel) and spreading area (lower panel) at various time periods (as indicated). Data represents means between cells from at least 3 independent experiments. Data are presented as mean ± SD. *P≤0.05; **P≤0.01; ***P≤0.001; ****P≤0.0001.

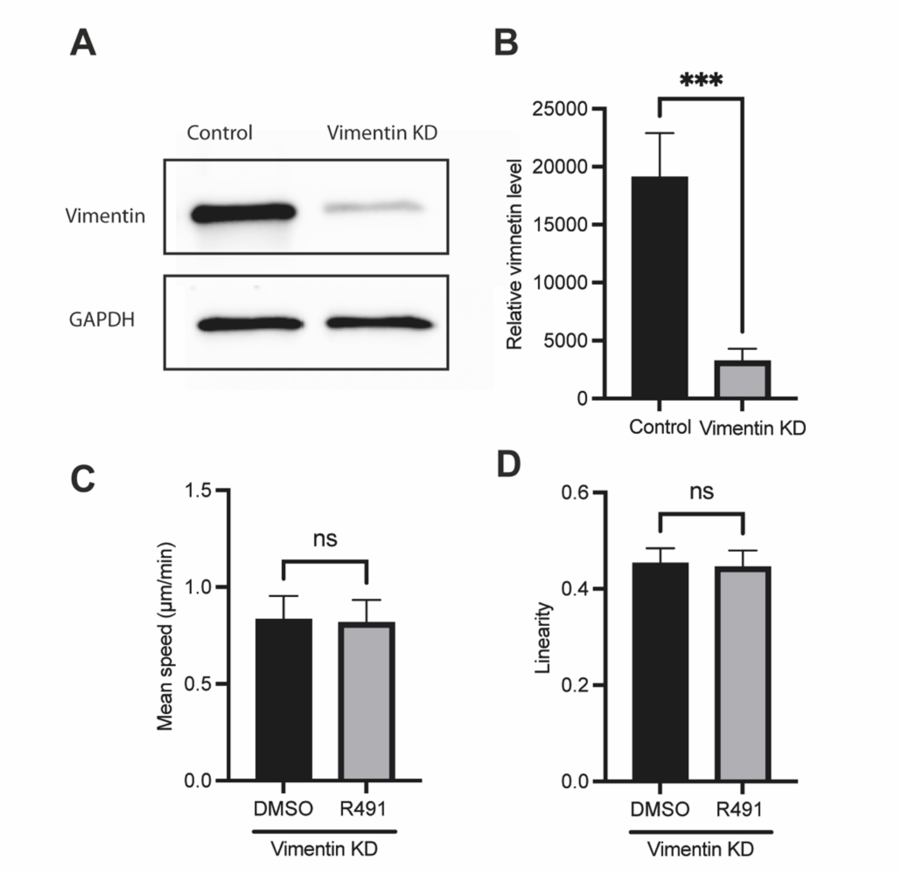


**Supplementary Figure 4.** Knock down of vimentin in BJ-Ras cells abolishes the effect of R491 on cell migration speed and persistence. The western blot showing the efficiency of vimentin knock down with siRNA **(A)**, and the quantification as shown in the top left panel **(B).** The effect of R491 in cell migration speed and linearity after the vimentin knock down **(C** and **D).** Graphs show means of at least 3 biological repeats. Data presented as mean ± SD. ***P≤0.001

**Supplementary Table 1: FRAP data with 95% confidence intervals.**
17 regions were removed from the whole data set due to either that the cell moved out of focus or the ROI was incorrectly positioned at the start of imaging.

| | Mock treated (n=21) | Drug treated 1µM (n=23) | Mock treated (n=14) | Drug treated 5µM (n=13) | Mock treated (n=17) | Drug treated 10µM (n=15) |
|---|---|---|---|---|---|---|
| Half-life (in secs) and 95% confidence intervals | 95.11 (81.73 to 111.0) | 90.03 (76.13 to 106.7) | 63.57 (53.00 to 75.93) | 75.6 (59.00 to 96.26) | 79.13 (59.87 to 104.2) | 59.06 (45.39 to 75.49) |
| y[max] and 95% confidence intervals | 0.730 (0.670 to 0.770) | 0.757 (0.720 to 0.800) | 0.63 (0.60 to 0.66) | 0.40 (0.38 to 0.43) | 0.72 (0.666 to 0.778) | 0.52 (0.492 to 0.553) |
| Rate of recovery (K) (in secs) and 95% confidence intervals | 0.0073 (0.0062 to 0.0085) | 0.0077 (0.0065 to 0.0091) | 0.011 (0.009 to 0.013) | 0.0092 (0.0072 to 0.012) | 0.0088 (0.0067 to 0.012) | 0.012 (0.0092 to 0.015) |
| The curve goodness of fit values: | | | | | | |
| Degrees of Freedom | 1048 | 1145 | 698 | 648 | 834 | 748 |
| R squared | 0.4003 | 0.3224 | 0.3311 | 0.2681 | 0.1917 | 0.1843 |
| Sum of Squares | 47.33 | 74.21 | 32.22 | 14.66 | 88.29 | 45.91 |

Comparing samples with the extra sum of squares F test:

| Comparing rates of recovery: | | | |
|---|---|---|---|
| | Mock (n=21) vs. Drug 1µM (n=23) | Mock (n=14) vs. Drug 5µM (n=13) | Mock (n=17) vs. Drug 10µM (n=15) |
| P value | 0.6388 | 0.3188 | 0.1316 |

Supplementary Material

| | | | |
|---|---|---|---|
| F (DFn, DFd) | 0.2204 (1, 2193) | 0.9948 (1, 1346) | 2.276 (1, 1582) |
| Shared K (rate) | 0.0075 (0.0067 to 0.0084) | 0.01052 (0.009171 to 0.01211) | 0.009918 (0.008232 to 0.01201) |
| Shared Half-time | 92.33 (82.40 to 103.6) | 65.86 (57.26 to 75.58) | 69.89 (57.71 to 84.20) |
| Degrees of Freedom | 2194 | 1347 | 1583 |
| R squared | 0.3567 | 0.4205 | 0.2161 |
| Sum of Squares | 121.6 | 46.92 | 134.4 |

| Comparing y[max]: | | | |
|---|---|---|---|
| | Mock 1µM (n=21) vs. Drug 1µM (n=23) | Mock 5µM (n=14) vs. Drug 5µM (n=13) | Mock 10µM (n=17) vs. Drug 10µM (n=15) |
| P value | 0.337 | <0.0001 | <0.0001 |
| F (DFn, DFd) | 0.9223 (1, 2193) | 41.28 (1, 1346) | 35.54 (1, 1582) |
| Shared y[max] | 0.746 (0.72 to 0.775) | 0.618 (0.595 to 0.643) | 0.657 (0.622 to 0.696) |
| Degrees of Freedom | 2194 | 1347 | 1583 |
| R squared | 0.3565 | 0.4031 | 0.1997 |
| Sum of Squares | 121.6 | 48.32 | 137.2 |
